# Big Data and Public-Private Partnerships in Healthcare and Research: The Application of an Ethics Framework for Big Data in Health and Research

**DOI:** 10.1007/s41649-019-00100-7

**Published:** 2019-09-30

**Authors:** Angela Ballantyne, Cameron Stewart

**Affiliations:** 1 University of Otago, Wellington, New Zealand; 2 The University of Sydney Law School, Sydney, Australia

**Keywords:** Public-private partnerships, Big data, Health information, Social licence, Bioethics, Stewardship, Transparency

## Abstract

Public-private partnerships (PPPs) are established to specifically harness the potential of Big Data in healthcare and can include partners working across the data chain—producing health data, analysing data, using research results or creating value from data. This domain paper will illustrate the challenges that arise when partners from the public and private sector collaborate to share, analyse and use biomedical Big Data. We discuss three specific challenges for PPPs: working within the social licence, public antipathy to the commercialisation of public sector health data, and questions of ownership, both of the data and any resulting intellectual property or products. As a specific example we consider the case of the UK National Health Service (NHS) providing patient data to Google’s DeepMind AI program to develop a diagnostic app for kidney disease. This article is an application of the framework presented in this issue of ABR (Xafis et al. 2019). Please refer to that article for more information on how this framework is to be used, including a full explanation of the key values involved and the balancing approach used in the case study at the end. We use four specific values to help analysis these issues: public benefit, stewardship, transparency and engagement. We demonstrate how the Deliberative Framework can support ethical governance of PPPs involving biomedical big data.

## Background

Realising the potential of Big Data requires an environment for trusted data sharing across multiple sectors. This domain paper will illustrate the challenges that arise when partners from the public and private sector collaborate to share, analyse and use biomedical Big Data. Here we demonstrate how the Deliberative Framework can support the ethical governance of such public-private partnerships (PPPs).

We focus on data staying within the health sector but moving between the private and public sectors. This can happen in a range of ways, for example:

- Private sector organisations can apply to use public sector data for research
- Electronic health records can include data generated in the private sector (apps, private hospitals, private specialist providers) and data produced by public health agencies
- Public and private sector agencies may form partnerships to pool resources and/or expertise to provide clinical care, or support research, innovation and product development

The data ecosystem is fragmented and complex, with health data increasingly being re-used, re-purposed, linked and shared in novel ways. On the one hand, regulation and legislation (such as the European Union’s General Data Protection Regulation) will apply equally to those managing health data in the public and private sectors. On the other hand, drivers, standards and reputational concerns for private sector actors are likely to be different from those of many public institutions. This diversity can prove challenging for PPPs using health data and seeking to traverse different expectations in the public and private sector. PPPs in Big Data will have to successfully navigate these potentially conflicting standards, norms and expectations. In areas of high-speed change, such as information technology and data science, the normative standards may not be well-established or codified; meaning partners are working in an opaque space and having to guess at data subjects’ expectations.

Collaboration between public and private sectors in Big Data in biomedicine raises some specific ethical issues for the Framework to consider. Working within the *social licence* requires aligning data initiatives with the reasonable expectations of the public. The social licence of public sector data use may not extend to private sector use. Empirical research consistently suggests public discomfort with the use of health data for *commercial gain*, or with commercial (for-profit) companies accessing their health data (Ipsos MORI 2014; Xafis 2015; Trinidad et al. 2012) Finally, PPP raise issues regarding ownership and control—both in relation to the raw data and the outputs of the partnership.

### Public-Private Partnerships

In many countries, PPPs are well-established components of the health system. A PPP is a cooperative arrangement between two or more public and private sectors parties. PPPs can vary widely in terms of the nature of the contract, risk allocation, funding arrangements and transparency requirements. They can range from project-specific collaborations to long-term strategic alliances between groups, to complex multi-consortia. The parties involved in a health PPP could include hospitals, primary care practices, specialist health providers, pharmaceutical and medical device companies, IT companies, non-governmental organisations (NGOs), private health insurers and construction firms (Yildirim et al. 2016). Partnerships are justified on the assumption that the partners can achieve results together which neither party could achieve alone nor achieve these results more efficiently (with respect to time or expense).

### PPPs in Big Data

PPPs are being established to specifically harness the potential of Big Data in healthcare and can include partners working across the data chain—producing health data, analysing data, using research results or creating value from data.

An early example was the establishment of the SNP Consortium in 1999. This was a global collaboration of private companies and public institutions working together to produce a map of single nucleotide polymorphisms (SNPs) in the human genome (Thorisson and Stein 2003). The SNP Consortium is broadly regarded as a success, exceeded its initial time targets and eventually mapped 1.25 million SNPs. An innovative protective patenting policy was adopted, where intellectual property rights were sought as a means to prevent other scientists from patenting the SNP data once they were publicly released (Cook-Deegan and Heaney 2010; Verbeure 2009). A key lesson here is the ability to agree common interests across the public and private sectors—both sectors wanted easy access to the SNP data for future research, and neither wanted research to be encumbered by having to negotiate with multiple patent holders to gain access to different bundles of SNPs for future research. A second lesson is that the accessibility of data for research (in this case the SNP repository) depends on *how* intellectual property rights are executed. This patenting strategy, designed to ensure that data remained protected in the public domain, is a counterexample to the sometimes knee-jerk reaction that all patents equate to a reduction in accessibility.

The European Union has an express commitment to supporting a “data-driven economy”; this includes a strategic focus on improving the use of Big Data in health research. The EU has funded a number of initiatives to bring public and private organisations together to share data and support innovation. One such project was Etox^1^, which united multiple pharmaceutical companies and several public partners to share data from pre-clinical in vivo toxicity research and produce a data pharmaceutical toxicology database. This database allows researchers to use data modelling to better predict which compounds will be toxic to patients and to remove these from the drug development pathway (Yildirim et al. 2016). The European Union has launched a new project called Public Private Partnerships for Big Data. It will build on lessons from national data sharing initiatives (such as France’s Terelab and the UK’s Open Data Institute) to establish “Innovation Spaces (i-Spaces)” (European Commission 2014). This will offer a purportedly secure environment for cross-sector collaboration and experimentation using both commercial and public data. In addition to technical platforms such as i-Spaces, the PPP will fund legal research to address barriers to data sharing in the EU—including work on data portability, ownership and the role of intellectual property. This example illustrates the diversity of PPPs arising in relation to biomedical data, including not just sharing data but also collaborating on data innovation pathways and legal research.

## Key Issues

In this domain paper, we will consider the specific challenges of social licence, commercialisation and ownership as they apply to biomedical Big Data PPPs.

### Social Licence

In areas of rapid change such as data science, practice can quickly outstrip the regulatory framework. This can result in data sharing that is within the parameters of the law, but may nevertheless present challenging ethical issues and/or be outside the social licence. Whereas law prescribes what agents *can* do and ethics prescribes what agents *should* do, social licence describes whether a given data use is accepted by stakeholders (Ballantyne 2018).

Working within the social licence has both intrinsic and instrumental values. Data subjects and those likely to be affected by Big Data initiatives have relevant interests that need to be considered and addressed. Data initiatives that exceed the social licence may experience public backlash and resistance (see for example the public rejection of the care.data project in the UK; Carter et al. 2015).

Social licence applies to industries, institutions or occupational groups *broadly*. Social licence permits some measure of flexibility in relation to common or expected modes of behaviour “and is used by the professions to claim a broad legal, moral and intellectual mandate” (Dixon-Woods and Ashcroft 2008). Failure to align a PPP data initiative with the social licence can have broad implications for public trust well beyond the specific PPP. A social licence breach could affect trust in the health research enterprise, in data sharing in general and/or in patients’ comfort in disclosing health information in the clinical context. Researchers often find regulatory and governance processes burdensome and an impediment to data innovation. But it is important to recognise that processes and institutions that reflect trustworthiness—such as transparency, regulation and good governance—can support a strong social licence for research.

Social licence granted for data use in the public sector will not automatically extend to data sharing with the private sector. This is because the activity in these sectors is subject to different (though sometimes overlapping) drivers. For example, there are reasonable expectations that public agencies will protect the public interest, use data to promote public benefit and demonstrate transparency. Private companies may share these goals,^2^ but they will also be motivated by innovations that offer a competitive advantage, protecting commercial secrecy and returning profit for shareholders. There may be various ways in which the interests of public and private parties diverge and these conflicts of interests within a PPP will need to be carefully navigated (see Lipworth (2019) for a discussion of conflicts of interest).

The data ecosystem is disjointed and there is considerable variability of standards, codes of practice and professional regulations in different sectors. Any PPP needs to consider at the outset the purpose for which the data was originally collected and the values and expectations underpinning action in this sector (be that health, immigration, education, or within the private or public sector) (Laurie and Stevens 2016).

### Commercialisation

One aspect that requires particular consideration in PPPs is the commercialisation of data in the private sector. Research consistently demonstrates significant public reluctance to allow commercial players access to health data (Grant et al. 2013). In response, some data access policies limit access by commercial entities (Shabani and Borry 2016).

A nuanced understanding of the difference sources of public antipathy to commercial access to health data is necessary to generate specific responses that address concerns and manage risks. Recent research from Ipsos MORI found that the general reluctance to allow commercial players access to health data was driven by a wide range of different concerns (Ipsos MORI 2016). Engagement with relevant stakeholders can help PPP partners understand and address the specific reservations and concerns of communities affected by PPPs for biomedical Big Data.

### Ownership

One concern with PPPs is that they provide private partners the chance to appropriate public datasets or extract undue value from their access to public data. This raises questions of ownership of raw data and ownership of research outputs.

The language of “ownership” is apparent in media, policy and bioethics debate about the appropriate management of health data. Sometimes ownership claims reflect a call for property rights in data. But the language of ownership can also act as a metaphor reflecting multiple concerns about current Big Data use. Concerns include the distribution of financial gain, control of the data, research and outputs, as well as recognition and reputation. Ownership claims can call attention to potential data harms relating to confidentiality, privacy, fairness, lack of transparency and accountability, as well as concern about the monetarisation of data. Notions of ownership are also relevant to debates about data portability, digital identities and data self-governance. So not all ownership claims should be interpreted prima facie as property claims.

How would a legal model of property apply to data? The legal concept of ownership is ill-defined, primarily due to the common law’s tendency to base property rights around possession. Ownership indicates the relationship between a person and a corporeal or incorporeal legal object. Property describes a bundle of rights to enjoy, use possess, dispose of and alienate a “thing” as well as the capacity to ward of any encroachment on the thing. Applying property models to something intangible, inexhaustible and fungible like data is challenging. Where the law does deal with property in data it takes a diverse approach depending on the context—for example, intellectual property, the doctrine of breach of confidence (Lord Advocate v Scotsman 1989; Stuckey 1981) and trade secrets (TS & B Retail Systems Pty Ltd v 3Fold Resources Pty Ltd 2007; Re Keene 1922; Morison v Moat 1851). Again, this makes it difficult to draw general principles of property of biomedical big data in PPPs.

For these reasons, we should avoid interpreting all ownership claims as property claims. We should think more laterally about what work the metaphor of ownership is doing in relation to PPPs in biomedical big data, and what solutions are available to ensure that PPPs can responsibility produce the benefit they are designed to create.

## Relevant values

The majority of the substantive and procedural values in this Framework apply to biomedical PPPs. In particular privacy, consent and conflicts of interest are relevant but these have been addressed in other papers in this Special Issue (Xafis et al. 2019; Xafis and Labude 2019; Lipworth 2019). Laurie (2019) contains relevant discussion of the importance of demonstrating a reasonable likelihood of *public benefit* and considers the importance of the values of *transparency* and demonstrating *trustworthiness* due to the diversity of actors involved in cross-sector data use.

The Deliberative Framework can help clarify which ethical values are engaged by PPPs and show how these tensions might be successfully navigated. Here we use the substantive values of public benefit and stewardship, as well as the procedural values of transparency and engagement to make sense of central ethical tensions in regard to PPP using biomedical big data.

### Substantive

Public benefit - partners must articulate how the PPP will produce public benefit (for example, development of data research capacity, new knowledge or understanding, and/or new data research methodologies) and how these benefits will be disturbed within and across communities.

Stewardship—data stewards have the dual responsibility to ensure that health information can be accessed and used appropriately, whilst also protecting privacy and demonstrating trustworthiness.

### Procedural

Transparency—being transparent can help strengthen the social licence for data sharing within PPPs, justify public confidence in institutions, ensure accountability and facilitate public debate; and can include sharing information about the proposed data uses, expected benefits, harm-minimisation strategies, degree of security and encryption, research results and/or the new algorithms.

Engagement—partners should engage with communities and stakeholders over the course of the project to determine their expectations regarding data uses and the parameters of the social licence; engagement activities should be proportional to the nature and size of the PPP.

We present a case study to illustrate some of these ethical challenges.

## Case Study: the National Health Service Data Sharing Agreement with DeepMind (Google)

As a specific example, we consider the case of the UK National Health Service (NHS) providing patient data to Google’s DeepMind AI program to develop a diagnostic app for kidney disease (Hawkes 2016). In 2015, the NHS provided Google DeepMind with 1.6 million identifiable and complete medical records (including sensitive information regarding, for example, abortion, drug overdoses, mental health and HIV status) to test a smartphone app called Streams that could help detect people with acute kidney disease. Google DeepMind is an artificial intelligence division within Google, which was created after Google bought University College London’s DeepMind in 2014. One of the justifications for DeepMind joining Google in 2014 was the potential to use Google’s scale and experience to achieve rapid progress in AI health interventions. Up to 25% of kidney deaths may be preventable if detected early, so DeepMind planned to use the NHS data to develop an algorithm to spot early signs of the disease and thereby save lives. This data sharing arrangement was controversial and elicited critique from multiple sources on various different grounds:

- The data sharing arrangement was outside the *provision of direct patient care* and therefore *specific patient consent* should have been sought, Fiona Caldicott, the UK National Data Guardian (Hern 2017).
- “Patients would not have *reasonably expected* their information to have been used in this way, and the Trust could and should have been far more transparent with patients as to what was happening.” Elizabeth Denham, UK Information Commissioner (Information Commissioner’s Office 2017).
- “All the *value is in the data* and the *data is owned by the UK taxpayer*. There has to be really serious thought about protecting those interests as we go forward.” John Bell, UK life sciences industry review (Delvin 2017).
- “…highlights the potential of the NHS, which we are not currently *capitalising* on. We must use the NHS as an engine for innovation.” Prof Sir Robert Lechler, president of the Academy of Medical Sciences (Laurie and Stevens 2016).

These quotes illustrate various concerns expressed by key thought leaders in regard to the NHS-DeepMind partnership, including: the lack of patient consent for data use, patients’ reasonable expectations regarding how public data will be managed, transparency, questions of ownership of public data, and the potential commercialisation of public health data. Of particular interest is the emerging idea that *public* big data repositories (such as the NHS) should be conceptualised as valuable *assets* for driving research and innovation.

## Application of the Deliberative Balancing Approach

Here we draw on four principles from the deliberative framework and show how these can be applied to the case study of the NHS-DeepMind partnership. Two distinctive substantive values that can help guide the decision-making around PPPs involving big data are public benefit and stewardship.

### Public Benefit

Public sector partners in particular must consider how the PPP will promote public benefit—as distinct from (only) facilitating commercial, political or departmental imperatives. For example, in the NHS-DeepMind partnership, one concern was that the UK taxpayers were not sufficiently or fairly rewarded for their contribution to the partnership. Bell argued that all the value was in the NHS data and therefore suggested that apps such as Streams (resulting from PPPs that rely heavily on access to public data) should be public property or at least co-owned so that the some of the profits are directed back to the public health system that enables their development. An alternative arrangement is to ensure the licensing agreement provides for free NHS use of the app.

Broadly, there are three forms of benefit that can arise from PPPs in big data: development of data research capacity, new knowledge or understanding, and/or new data research methodologies (coding and algorithms). Specifically, public benefits might include new medical innovations, better understanding of health pathways, strengthened R&D sector, increased financial investment and strengthened economy. A PPP needs to articulate which public benefits are reasonably likely to accrue and how these will be measured and evaluated. It is important to note that these benefits are unlikely to be evenly shared across the community; and so issues such as exacerbating health inequalities, equity and fair distribution of benefits must also be considered.

We mentioned above the innovative use of intellectual property rights in the SNP consortium. A key insight from this example is that access to research results depends on how intellectual property rights are used and enforced. There are many other examples of creative strategies, used by private and public sector players, to advance the open science agenda and ensure that the benefits of research remain in the public domain, e.g.:

- the Merck and the US Department of Energy funded project to ensure Expressed Sequence Tags (EST) sequencing remained in the public domain;
- the Bermuda Rules facilitating the rapid sharing of genomic sequencing data; and
- the International HapMap project commitment to keeping result in the public domain. (Cook-Deegan and Heaney 2010)

These solutions require multi-level strategies—identification of common interests between public and private partners, attention to the underlying values of the activity (and the relevant social licence), articulation of the public benefit, engagement with the research community, transparency and openness regarding strategies and the underlying reasoning.

### Stewardship

The NHS is a steward of the UK public’s health information. They have the dual responsibility to ensure that health information can be accessed and used appropriately, but also of protecting privacy and demonstrating trustworthiness. Stewardship of a public resource such as national health dataset needs to take account of the publics’ reasonable expectations regarding data use.

Community engagement, transparency and due process in decision-making can help demonstrate that public sector agencies are trustworthy stewards of health data, even when there is a lack of consensus about the specific data sharing project. Essential to genuine public debate is the requirement that dissenting voices are heard and recorded, that the justification for disregarding dissenters are reasonable and publicly accessible and that decisions can be reviewed and evaluated over time. The PPP could consider establishing a joint governance body or ethics committee to guide the data sharing process (stewardship is also discussed in detail in Laurie (2019)).

Two distinctive procedural values that can help guide the decision-making around PPPs involving big data are transparency and community engagement.

### Transparency

Transparency can help justify public confidence in institutions, strengthen the social licence, ensure accountability and facilitate public debate. Transparency in big data PPPs might include making the data uses, expected benefits, harm-minimisation strategies, degree of security and encryption, research results, and coding/algorithms accessible to others outside the team and/or open to the public.

A potential barrier to transparency in relation to PPPs is the commercial and reputational concerns of both public and private partners—for example, public institutions may be under political pressure not to release information about projects that could be politically contentious and private companies may claim commercial sensitivity in relation to research processes or outputs. Neither reputational concerns nor commercial interests are alone sufficient to outweigh the value of transparency in how PPP initiatives are conducted. Arguments in favour of public transparency and openness are especially compelling when PPPs involve sharing population health data sets, collected in public systems, funded by tax resources, and when PPPs are defended on the grounds of public benefit. Partners should consider if these features apply to a proposed PPP.

### Engagement

In pluralistic, multicultural societies, there will be different views on acceptable data use. The parameters of the social licence will change over time as citizens became more comfortable with, or more suspicious of, various uses of Big Data. This requires PPPs, especially those operating over the medium to long term, or using large public database to continue to engage with communities and stakeholders as projects progress. As noted above, failure to engage effectively with the public and patients about PPPs can result in withdrawal of the social licence for activities that extend beyond the specific PPP.

Partners to a proposed PPP should consider the purpose for which the data was originally collected and the relevant values and expectations underpinning activity in the sector. In this case, the NHS should consider whether patients would reasonably expect that their clinical data was being shared with Google, and whether novel transparency and engagement strategies can help to manage patients’ expectations regarding data sharing.

Key challenges in relation to patient and public engagement are *which* patients to engage with and *how* best to engage. Patient engagement can over-rely on the well-connected and well-informed, patients who are unable to offer useful input (for example not well-trained on technical aspects such as data security strategies), and/or reliance on patient advocacy organisations (who may experience COIs due to industry funding) (Largent et al. 2018).

In some cases, the data use will disproportionately affect certain groups (for example, demographic, geographical, ethnic, disease specific) and this provides an indication of appropriate patient groups to engage. However, value of the NHS dataset is in its breadth. It is not clear that specific groups would be disproportionally affected and therefore candidates for engagement.

One thing for the NHS to consider is that the most vulnerable and disadvantaged members of society often rely on multiple government services, and often have high health needs (and may well generate more health data). But, by virtue of this same vulnerability, are also likely to have the least capacity to influence the data sharing practices and public debate. The paradox is that those with most at stake may have least capacity to exert influence (see the discussion on vulnerability in Xafis et al. (2019)). In large population datasets (including data from many different patient groups), priority could be given to engagement with some of the more vulnerable patient groups.

## Balancing Approach

In order to demonstrate how the balancing approach can help clarify and resolve ethical disputes, we focus on one specific element of this case: *did the NHS behave as responsible data stewards?*

Stewardship involves promoting appropriate use of the data, whilst also safeguarding data subjects’ interests. These twin objectives can result in a clash between maximising use of the data, especially where this results in some public benefit, and ensuring transparency, public engagement and good governance, which can delay or restrict access to the data. Accelerating the development of the Streams app was a primary goal of the PPP. One striking feature of this PPP was the speed with which the parties moved from discussion to data transfer. This propelled the development and clinical roll-out of Streams. However, there were serious concerns that appropriate consultation and engagement processes were not followed, and that governance structures were lacking. The widespread criticism of this PPP, from diverse sectors, suggests a lack of sufficient public justification for the terms of the data sharing agreement, including for example an explanation for the need to share identifiable health data. Governance and engagement processes could have included consultation with the National Data Guardian, research ethics review for access to identifiable health data, privacy impact assessment, public notification and opt-out processes for dissenting patients. Data sharing proceeded without consent or research ethics committee approval. Robust governance structures may delay the speed of innovation, but they can demonstrate trustworthiness and can sustain the social licence for PPPs.

Given the NHS data is a public resource, “appropriate use” would require that the PPP produce sufficient public benefit. The Streams app is a successful product, which according to clinicians who use it has improved identification of AKI in UK hospitals. This offers a direct health benefit to UK patients and cost-savings for UK tax payers. However, additional mechanisms for ensuring public benefit, such as co-ownership of the app and profit-sharing appear not to have been part of the PPP. DeepMind has now indicated that they plan to initiate a global scale up of Streams, along with other AI tools developed and tested using NHS data (Hassabis et al. 2018). A slower negotiation process, with broader UK stakeholder input, may have resulted in joint intellectual property rights for the NHS.

## Conclusion

The deliberative framework is designed to help potential partners identify the relevant values and interests at stake in PPPs and see where these might clash—maximising data use for research and innovation may exceed the limits of the social licence and not allow time for sufficient public engagement. In different contexts, priority will appropriately be given to different values. This process will allow PPPs to articulate the ethical trade-offs between values, support deliberation about appropriate data sharing, and communicate their reasoning to stakeholders.

## Footnotes

1
http://www.etoxproject.eu/

2 In addition, some private companies committee to triple bottom line accounting, where they hold themselves responsible for social and environmental results, in addition to financial results.
